# The cohort multiple randomized controlled trial design was found to be highly susceptible to low statistical power and internal validity biases

**DOI:** 10.1016/j.jclinepi.2017.12.008

**Published:** 2018-03

**Authors:** David Reeves, Kelly Howells, Mark Sidaway, Amy Blakemore, Mark Hann, Maria Panagioti, Peter Bower

**Affiliations:** a NIHR School for Primary Care Research, Manchester Academic Health Science Centre, University of Manchester, Oxford Road, Manchester, M13 9PL, England; b Salford Royal NHS Foundation Trust, Salford Royal Foundation Trust, Stott Lane, Salford, M6 8HD, England; c NIHR Greater Manchester Primary Care Patient Safety Translational Research Centre, Manchester Academic Health Science Centre, University of Manchester, Oxford Road, Manchester, M13 9PL, England

**Keywords:** cmRCT, Trials within Cohorts, Sample size calculation, Bias, Methods, Cohort study

## Abstract

**Objectives:**

The “cohort multiple randomized controlled trial” (cmRCT) is a recent innovation by which novel interventions are trialed within large longitudinal cohorts of patients to gain efficiencies and align trials more closely to standard clinical practice. The use of cmRCTs is outpacing its methodological understanding, and more appropriate methods for designing and analyzing such trials are urgently needed.

**Study Design and Setting:**

We established the UK Comprehensive Longitudinal Assessment of Salford Integrated Care cohort of 4,377 patients with long-term conditions within which we are conducting a cmRCT (“Proactive Telephone Coaching and Tailored Support”) of telephone-based health coaching.

**Results:**

We identify some key methodological challenges to the use of the cmRCT in actual practice. Principal are issues around statistical power, sample size, and treatment effect estimation, for which we provide appropriate methods. Sampling procedures commonly applied in conventional RCTs can result in unintentional selection bias. The fixed data collection points that feature in cmRCTs can also threaten validity.

**Conclusion:**

The cmRCT may offer advantages over conventional trial designs. However, a cmRCT requires appropriate power calculation, sampling, and analysis procedures; else, studies may be underpowered or subject to validity biases. We offer solutions to some of the key issues, but further methodological investigations are needed. Cohort multiple RCT–specific Consolidated Standards of Reporting Trials guidance may be indicated.

**What is new?**

**Key findings**
• The cohort multiple randomized controlled trial (cmRCT) is a recent innovation in “efficient” trial design that is gaining in popularity. However, design and analysis of a cmRCT requires appropriate power calculation, sampling and analysis procedures, or such studies can find themselves underpowered or subject to selection and other validity biases.

**What this adds to what was known?**
• We identify key methodological challenges to the use of the cmRCT in actual practice. Principal are issues around statistical power, sample size, and treatment effect estimation. We also describe hitherto unidentified validity risks inherent in the design, such as sampling practices commonly applied in pragmatic trials, which when applied to a cmRCT can result in selection bias, and validity issues related to the fixed data collection points that feature in cmRCTs.
• We provide appropriate methods for power calculation and show that unless levels of participant eligibility and consent are substantial, the sample size requirements for a cmRCT may be impracticably large.

**What is the implication and what should change now?**
• Pilot studies are essential to determine likely rates of eligibility and nonconsent for the purposes of cmRCT sample size estimation.
• It is important that trials using the cmRCT design publish sufficient detail on their processes, along with summary statistics, to reassure users of the research that the validity threats specific to this design have been appropriately addressed. Cohort multiple RCT–specific Consolidated Standards of Reporting Trials guidance may be advised.

## 1 Introduction

Randomized trials are fundamental in evidence-based medicine but often struggle to recruit, leading to problems in both internal validity (especially power) and external validity. There is widespread interest in the development of innovative trial designs that can more effectively recruit and retain patients and make trials more efficient and patient centered. One such innovation is the “cohort multiple randomized controlled trial” (cmRCT) [1], a form of “Trials within Cohorts” (TwiCs) design in which novel interventions are trialed within much larger, typically longitudinal cohorts of patients to take advantage of potential recruitment, cost, and other efficiencies [2].

It is claimed that the cmRCT design can overcome many of the shortcomings of traditional pragmatic randomized controlled trials (pRCTs) [1]. Under a pRCT potential participants are provided with information about the trial and the available interventions, and consenting participants are then randomized between trial arms. All patients are told about the different treatments in the trial arms, including any new treatment, but only half are randomized to that new treatment. Patients who have a strong preference for a particular treatment (on offer within the trial or outside of it) or who are concerned about the risk of being randomized to an unproven treatment may be less likely to agree to participate [3]. Even among participants, there is a risk that randomization to the nonpreferred arm may cause dissatisfaction affecting withdrawal and outcome reporting [3], [4].

In contrast, the cmRCT design aims to make the trial consent procedure more like standard health care, where people are only asked to consent to treatments they are being offered and are not told about treatments they cannot access. Under this design, a substantial cohort of participants is first established and then followed up at regular time intervals. To conduct a cmRCT of a new intervention, all cohort participants eligible for the treatment are first identified and then a random sample selected and offered the treatment, which they can either consent to receive or decline. All remaining eligible patients—that is, all patients eligible for the treatment but not offered it—constitute the control arm. These patients are not informed about the trial or the randomization, so they never hear about treatments that they will not receive. Relevant outcomes and other measures are taken on all patients in both arms as part of the regular follow-up process. Further cmRCTs of other interventions can be conducted within the same core cohort of patients.

Advocates of the cmRCT design claim significant advantages regarding recruitment, patient centeredness, and efficiency including costs. Enhanced recruitment stems from basing the trial within an established cohort and from the simplified consent process, which offers a straightforward choice between agreeing to the experimental treatment or not; while disappointment bias and cross-over may be reduced by eliminating randomization to the control arm. Efficiencies can be gained by conducting multiple RCTs within the same cohort, while the availability of large numbers of potential controls allows the number offered treatment to be reduced without loss of statistical power, thus saving treatment costs. Since the design was first proposed, a number of patient cohorts and related cmRCTs have been established [5], [6], [7], [8], [9], [10], [11], [12], [13], [14]; however, very few of these have yet reported, and good evidence to support these claims is lacking. We conducted a search for articles reporting the results of cmRCTs and found only two that have reported actual recruitment figures [15], [16]. In a small pilot cmRCT of a homeopathic treatment for menopausal hot flushes, 17 of 24 women accepted the offer of treatment (71%) [15]. The Depression in South Yorkshire (DEPSY) trial achieved 40% consent to treatment (74/185) and reported that recruitment was more efficient and overall attrition smaller than other depression trials [16]. However, differential attrition was high, both between arms (13% among controls vs. 32% among intervention patients) and between intervention group patients who did (12%) and did not (66%) consent to treatment. No control arm participant crossed over to the treatment arm, but owing to nonconsent, 60% of the intervention group effectively crossed over to control. These results imply a complex mix of both benefits and disadvantages.

Many of the advantages claimed for the cmRCT relate to the use of prerandomization, by which eligible patients are randomized to trial arms first and consent sought only from those assigned to the experimental treatment. In this respect, the cmRCT is akin to the well-known and rather contentious Zelen's design [17], [18]. The key differences to the Zelen lie, first, in the incorporation of prerandomization into a framework for conducting RCTs using subsets of a large patient cohort and regular collection of outcomes data; and second, in the potential to conduct multiple trials within the same cohort, thus increasing efficiency.

The ethics of prerandomization have been much debated in the trials literature [19], [20], and in clinical settings, it is often regarded as unacceptable, not least because control participants are used as research subjects without their explicit consent [21]. However, proponents of the cmRCT have argued that the process of random selection from a cohort of people who gave consent at the outset for their data to be used for research is less ethically contentious than the Zelen design [1]. They also propose that ethical concerns might be further reduced by the use of a “staged” consent process, in which participants give consent on entry into the cohort for future allocation to any experimental treatment to be decided by chance [22]. However, this modification makes the consent process more extensive and complex than in the original design conception.

The cmRCT design is arguably most efficient when the cohort draws on an existing patient registry and routinely collected outcomes. However, the outcomes available might not be optimal and additional covariates, such as patient demographics, are limited. Such restrictions can be avoided by recruiting a cohort from scratch and collecting tailored measures, but this is a substantial undertaking, particularly when individual consent is required, and unless multiple cmRCTs are planned, could prove less efficient than dispensing with the cohort and directly running a Zelen-type trial or a standard pRCT. In addition, since the participants in any cmRCT will consist of treatment-eligible patients drawn from the cohort, generalizability of results will depend critically on the extent to which the cohort itself represents the actual target population.

In the process of recruiting a cohort and conducting a cmRCT ourselves, we have identified further, but less obvious, features of this design that raise important methodological issues of research design and analysis so far not addressed in the literature. In this article, we consider these issues and report how we tackled them within our own study and their implications for the design, conduct, and analysis of cmRCTs. We begin by describing the study, then discuss issues of statistical power, sample size, and treatment effect estimation, and finally address issues of practical concern around sampling procedures and follow-up scheduling.

### 1.1 The CLASSIC cohort and PROTECTS cmRCT

We implemented the cmRCT design in the context of a behavior change intervention in older people with chronic conditions. We first established the Comprehensive Longitudinal Assessment of Salford Integrated Care (CLASSIC) cohort: between November 2014 and February 2015, a questionnaire was mailed to 12,989 people aged ≥65 years with one or more chronic conditions identified from the lists of 33 participating family practices in the city of Salford, UK. Potential participants were also told that they would receive a similar questionnaire every 6 months or so and might be approached about related research projects. After a repeat mailing to nonresponders, 4,377 (34%) patients returned completed questionnaires; these make up the CLASSIC cohort. We are now conducting a cmRCT called Proactive Telephone Coaching and Tailored Support (PROTECTS) within this cohort, where the intervention involves telephone-based health coaching delivered in six monthly 20 minute calls, focused on lifestyle support around diet, exercise, smoking, alcohol, and low mood. A total of 1306 patients from the CLASSIC cohort met the eligibility criteria for PROTECTS, of two or more long-term conditions and lacking basic knowledge about self-management. (Further details of CLASSIC and PROTECTS are provided elsewhere [23], [24]).

### 1.2 Power and sample size

One key feature of a cmRCT is that all members of the cohort randomly selected into the treatment group must remain in that group in analysis, regardless of whether they subsequently consented to receive treatment or not. Removal of patients who declined treatment could potentially seriously bias the resulting effect estimate because patients who would have refused treatment if offered it undoubtedly also exist in the control group, but being unknown cannot be removed. This feature has several important implications for this type of trial, including the correct calculation of power and sample size, and even sampling procedures (see below).

This key point has been largely neglected by previous and existing cmRCTs, which have nearly all applied the same methods of sample size calculation as for a pRCT, under assumed 1:1 allocation to trial arms [5], [9], [14]. For PROTECTS, in the absence of guidance to the contrary, we likewise followed standard methods and, with the objective of having 80% power to detect a mean standardized treatment effect of 0.25 on any continuous outcome measure, estimated the required sample to be 504 or 252 per arm (assuming—based on previous pRCTs—an attrition rate of 25% and [conservative] correlation of 0.5 between baseline and follow-up outcome scores). We therefore randomly selected 252 eligible patients to be offered the intervention, with the remaining 1,054 constituting the control group. This gave PROTECTS many more control patients than the 252 indicated by the power calculation, which at face value might suggest excess power. However, in the event, only 100 of the 252 selected patients (40%) consented to receive the intervention which, for reasons explained below, in fact left PROTECTS greatly underpowered and led us to subsequently offer treatment to a further “top-up” sample of 252 patients, resulting in a final total of 207 consented patients and a consent rate of 41% (207/504).

The treatment arm of PROTECTS is thus a mixed group made up of 41% patients consenting to treatment and 59% not consenting. To avoid introducing bias, all patients in PROTECTS selected for treatment must remain in that group in analysis, and this results in a “dilution” of the mean difference between trial arms. Suppose that in PROTECTS, the mean effect in patients who consent to treatment is 0.5 and zero in nonconsenters. In consequence, the mean effect across all patients in the treatment arm will be 0.5 × 0.41 + 0.0 × 0.59 = 0.21. The same is not true of a pRCT, where nonconsenting patients are entirely excluded. Hence, when patient consent is less than 100%, the cmRCT design when compared with a standard pRCT, must detect a smaller overall mean difference between trial arms to reach statistical significance.

The impact of nonconsent on power and sample size in PROTECTS is explored further in Table 1. Notably, when consent is lower than 62%, the CLASSIC cohort does not contain sufficient eligible patients to detect a treatment effect of 0.25 at 80% power, no matter how many are selected for treatment. Given the actual sample size and consent rate of PROTECTS, the detectable effect of consent to treatment at 80% power is in fact 0.39. For comparison, Table 1 also shows the samples needed for a pRCT under the same set of assumptions. There is a sharp contrast: the figures show that it would still be possible to fully power a pRCT using the CLASSIC cohort with consent as low as 40%. However, at high consent rates—70% or more—the cmRCT design has a considerable sample size advantage, requiring offering treatment to less than half as many patients as a pRCT [2].

**Table 1:** Samples sizes required for PROTECTS to have 80% power to detect an effect of 0.25 in patients who consent to treatment, under both cmRCT and pRCT designs

Percentage of patients consenting to trial	cmRCT				pRCT			
	Effect size to be detected (adjusted for consent rate)	Patients selected	Patients consented	Patients in treatment vs. control arms	Effect size to be detected	Patients invited	Patients consented	Patients in treatment vs. control arms
100%	0.25	139	139	139 vs. 1167	0.25	504	504	252 vs. 252
90%	0.225	178	160	178 vs. 1128	0.25	560	504	252 vs. 252
80%	0.2	237	190	237 vs. 1069	0.25	630	504	252 vs. 252
70%	0.175	344	241	344 vs. 962	0.25	720	504	252 vs. 252
62%	0.155	653	405	653 vs. 653	0.25	813	504	252 vs. 252
60%	<80% power				0.25	840	504	252 vs. 252
50%	<80% power				0.25	1008	504	252 vs. 252
40%	<80% power				0.25	1260	504	252 vs. 252

*Abbreviations:* cmRCT, cohort multiple randomized controlled trial; pRCT, pragmatic RCT.

The implications of this for the powering of a cmRCT are examined in more detail below, after first considering exactly what the “effect” is that the design is estimating.

### 1.3 Treatment effect estimation

Some authors have described cmRCTs as producing an estimate of the intention-to-treat (ITT) effect (effect of accepting treatment [25] or effect of accepting and fully complying with treatment [26]), albeit an inherently biased estimate in the presence of nonconsent. However, in point of fact, the design compares all patients randomly selected to be offered treatment with those not selected, hence—ignoring for now issues of preselection into the cohort itself—the effect being estimated is best characterized as the mean effect of being selected for treatment, or assuming the percentage of noncontactable patients to be suitably low, the mean effect of the offer of treatment regardless of whether that offer is taken up or not.

By contrast, in a pRCT patients who do not give consent are entirely excluded from the trial, and the effect being estimated is that of randomization to treatment, or assuming small numbers decline on being informed of allocation, the mean effect of agreement to treatment, particularly under an ITT protocol.

Hence, in summary and approximately, a cmRCT provides an estimate of the mean effect in people offered treatment, whereas a pRCT provides an estimate of the mean effect in people agreeing to treatment. Both estimates will be more-or-less biased relative to the true population mean effects, depending on the mechanisms determining selection, consent, noncompliance, and attrition in each case, but the key point is that the two designs estimate different effects, which only converge at 100% consent to the cmRCT. This highlights the issues associated with powering cmRCTs using the standard methods used for pRCTs.

**Fig. 1:**
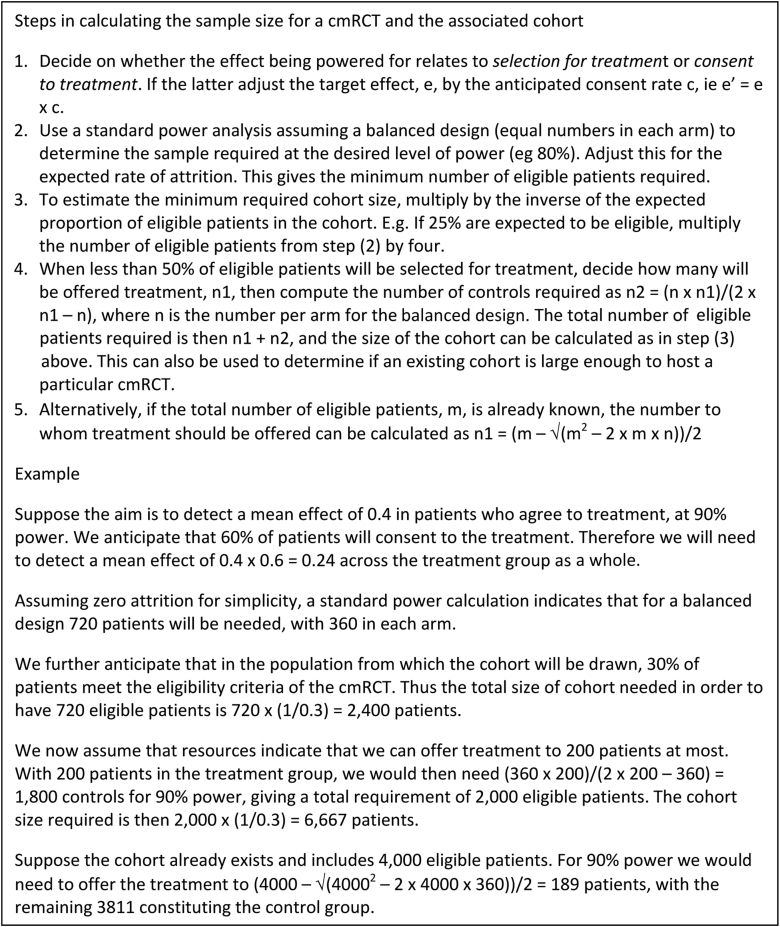
Steps in calculating sample size for a cmRCT. cmRCT, cohort multiple randomized controlled trial.

Power and sample size can be calculated using standard methods, but with some adaptations (Fig. 1). It is first necessary to decide whether the treatment effect being powered for relates to those offered treatment or those agreeing to treatment. The former applies where interest is in the mean effect at a population level, including all those who turn down the offer of treatment. The latter applies where the focus is patients who actually enroll into treatment. When the target effect pertains to those agreeing to treatment, this must be multiplied by the anticipated consent rate before use in the sample size calculation, to account for dilution of the overall mean effect from nonconsent. This will be the case, for example, when the target effect is based on the “clinically important change” for an individual patient. Since cmRCTs are typically unbalanced designs, with more control than intervention patients, an adaption for this in the sample size calculation is also required. Fig. 1 presents a procedure for this, along with methods for assessing the impact of varying the number of patients selected for treatment or the size of the cohort.

**Fig. 2:**
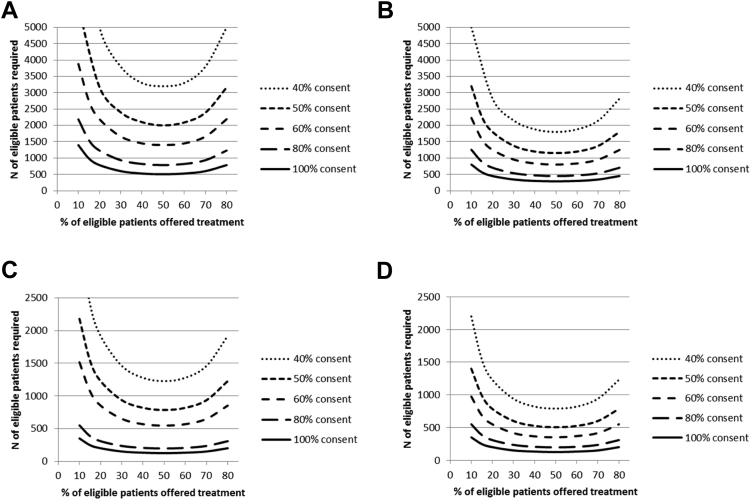
Numbers of eligible patients required for different percentages offered treatment and consent rates, for 80% power to detect a standardized treatment effect of: (A) 0.25; (B) 0.33; (C) 0.4; and (D) 0.5.

In any practical application, we advise calculating sample sizes under a range of assumptions about rates of eligibility and consent, and the number to be offered treatment, because the samples required can be very sensitive to changes in these parameters. Fig. 2 shows the numbers of eligible patients required in the cohort, assuming no attrition and 80% power, for various combinations of treatment effect size, consent rate, and percentage offered treatment. The graphs illustrate the considerable impact of the consent rate on the sample required, particularly when only a relatively small percentage of patients will be offered the treatment. Thus, it is vitally important to estimate the size of the required cohort at the research design stage and not to just assume that a cohort of around 1,000 to 3,000, say, will be sufficient for any planned cmRCT, as has often been the case [5], [6], [7]. An undersized cohort may not yield sufficient eligible patients to power the cmRCT.

In view of this impact of nonconsent on the estimate of effect, instrumental variable (IV) and complier average causal effect (CACE) methods have been proposed as means for estimating the mean effect for patients who consent to treatment only [25], [26], [27]. These methods generally produce larger effect estimates: CACE, for example, is the offer-of-treatment effect divided by the rate of consent [28] and will, for instance, double the estimate of effect when consent is 50%. It might, therefore, be supposed that CACE provides additional power to detect an effect, but this is not so because the standard deviation of the CACE will generally be greater by at least the same degree [29]. IV estimators behave in a likewise manner [25].

### 1.4 Sample selection

In a cmRCT, eligible patients not randomly selected into the treatment group are by definition members of the control arm. As a consequence, it becomes critically important when selecting the treatment group that all eligible patients have equal probability of being selected. If patients with certain characteristics are more likely to be selected for treatment than others, then such patients will ipso-facto be underrepresented in the control group, resulting in an imbalance between trial arms. This rules out any “over-sampling” of particular patient strata, for example, to compensate for smaller numbers. As an example, suppose that in a hypothetical cmRCT, the eligible patient group is 67% male and 33% female. The researcher may wish to compensate for fewer females by selecting at a ratio of 2:1 female to male. As a result, the treatment group will be 50% female, but the control group will be 28% female. Although this imbalance could be corrected for in analysis, this is far from ideal.

The same factor has important implications for other sampling procedures as well. Fifty-one of the 504 patients selected for PROTECTS could not be contacted despite multiple attempts, hence were never even offered the intervention. Despite this, these patients must remain in the treatment group throughout the trial, including final analysis: equivalent but nonidentifiable patients exist in the control group, hence to exclude them from the treatment arm would be to risk introducing bias. The same argument applies to patients subsequently found to be ineligible for the intervention or who withdraw from the trial, and in general, it seems that under the cmRCT design, it becomes inappropriate to exclude patients subsequent to selection for any reason.

Sampling complexities also impact on top-up sampling. Top-up sampling is a common strategy in pRCTs to boost recruitment, usually by extending the recruitment period or expanding the catchment area. However, under the cmRCT design, unless the cohort itself is going to be increased, the top-up sample will be limited to the subgroup of existing and so far unselected eligible patients. We have found, however, that to avoid introducing bias, top-up selection must be based on initial eligibility and not eligibility at the time of the top-up. In PROTECTS, we added a top-up of 252 patients after the 6-month data had been collected, but at this point, 30% of originally eligible patients had ceased to meet the eligibility criteria, while another 17% of the cohort had become eligible. Selecting the top-up on the basis of 6-month eligibility, therefore, looked attractive, but we realized that this would result in a different control group for patients selected at 6 months compared with those selected at baseline because of the cross-overs in eligibility. The presence of a different, but overlapping, control group for the top-up sample would then be highly problematic in analysis.

We therefore found it necessary to draw the top-up sample from the original pool of (previously unselected) eligible patients based on their initial assessments, rather than newer data. Furthermore, this had to include those we knew to be uncontactable or who had since withdrawn or moved away, who would otherwise automatically be assigned to the control arm, potentially biasing baseline comparability and impacting on imputation of missing data and subsequent analysis.

The above observations indicate that the cmRCT design carries a considerable risk of sampling procedures causing an unintended systematic difference between the treatment and control arms, adding bias to the resulting treatment effect estimate. In contrast, the pRCT design is far less sensitive to deviations from purely random sampling: under a pRCT, selected patients who consent to participate will subsequently be randomly allocated to the trial arms, which will therefore be balanced even if the characteristics of the patients consenting to the trial are not fully representative of all eligible patients.

### 1.5 Timing of treatment and follow-up

**Fig. 3:**
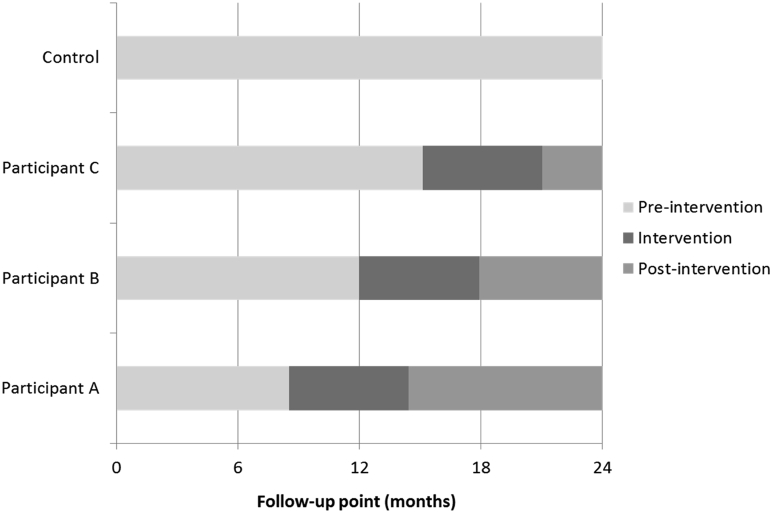
PROTECTS trial, outcome measurement points and timing of intervention for four example participants in PROTECTS. PROTECTS, Proactive Telephone Coaching and Tailored Support.

The cmRCT design proposes that outcome data are collected at fixed time intervals. But for practical and resource reasons, it is common for a trial to roll treatment out to participants over a period of time, such that the start and end points of treatment relative to the fixed measurement points can vary considerably between patients. Fig. 3 illustrates this for a few selected PROTECTS participants. Although some (e.g., participant A) were offered treatment shortly after the 6-month measurement point (no patient was offered treatment before 6 months), for others, the offer was not made until 12 months or later (B and C). This resulted in a very wide variation in delays between baseline measures and start of treatment—ranging from 259 to 513 days—and correspondingly wide variation in lengths of follow-up. Even if we were to “reset” the baselines to be each participant's measurement point immediately before treatment, substantial variation would remain, 34–182 days.

It is true that pRCTs can also experience variability in pretreatrment and posttreatment time durations, which is typically ignored in analysis. However, within a pRCT, it is usually possible to tailor the times of data collection to each individual participant and so minimize such variability. The fixed measurement points within a cmRCT do not allow the same degree of control. This raises the question of whether individually targeted measurement points might be possible under the cmRCT design. We cannot see how this could be done in a trial such as PROTECTS, where baseline measures were required to first identify the pool of eligible participants. A further complexity would be how to collect data on control participants—for whom there is no defined treatment period (Fig. 3)—in a way that ensures temporal equivalence between the trial arms.

## 2 Discussion

The cmRCT design is intended to address limitations of the pRCT by aligning trials more closely to actual health-care practice. However, the design raises many ethical, methodological, and practical issues, many of which are not immediately obvious (see “What is new?”).

Compared with a pRCT, a cmRCT first involves recruiting a large cohort and then regularly collecting outcome data on participants. The additional overheads associated with this activity can only be justified if the design has sufficient compensating benefits. One potential benefit is improved recruitment and compliance and reduced attrition. Convincing evidence for such benefits remains to be demonstrated. The rates in PROTECTS of 41% consent and, to date, 31% attrition are not suggestive of any dramatic improvement over traditional studies of telehealth interventions in people with chronic conditions [30], [31]. DEPSY—the only other full cmRCT to so far report—had a slightly better rate of consent but experienced much higher attrition amongst intervention arm participants than controls [16]. We did not ask participants before entry into the cohort for consent to be selected at random for the offer of future experimental interventions [22] but did inform patients that they may be asked at a later date if they are willing to help “test new ways of delivering services”. At the time of writing, our overall compliance rate of scheduled telehealth sessions was 78%. Unfortunately we lack compliance rates from comparable pRCTs to know whether this is a substantial improvement or not. Recruitment and compliance rates from other ongoing cmRCTs when they report will help establish a broader evidence base around participation.

Another potential advantage is that a cmRCT will generally require the experimental treatment to be delivered to a considerably smaller sample of patients than would be necessary for an equivalently powered pRCT—but only if the rate of consent to treatment is sufficiently high. As a rule of thumb, we suggest that a consent rate of at least 60%, and preferably 70%, is needed to make a cmRCT more efficient than a pRCT, though clearly other factors should also be taken into account. We strongly advise that any research group considering a cmRCT first conduct pilot work to gauge the likely rates of eligibility and consent in the full trial.

The cmRCT highlights issues around the effect being estimated. The design principally produces an estimate of the effect of the offer of treatment (or more strictly, selection for treatment). In some contexts, this may be the most appropriate estimate of effect. PROTECTS, for example, is a model of treatment designed to be proactive, using identification of patients in need to achieve population benefit, and—although we powered for the effect of consent to treatment—arguably the consent rate itself is a very relevant factor in the overall benefits (or otherwise) of the intervention. The cmRCT design is by its nature more suited to the estimation of a population-level effect than to the effect of treatment per se; however, we do question— as others have done [25]—whether consent under the conditions of a cmRCT is a reliable guide to take up once an intervention becomes available in routine practice.

In situations where researchers, clinicians, and policy-makers require information on the benefits of actual treatment, not just the offer of it, nonconsent must be accounted for in calculating sample size. Not to do so may well result in a considerable underestimate of the numbers needed and hence an underpowered trial. None of the existing cmRCT trials that we are aware of have adjusted the target effect size for nonconsent. While IV and CACE methods can be used to estimate the effect in patients consenting to treatment, these require additional assumptions and cannot compensate for the lack of power.

Our investigation shows that the consent and eligibility rates have a considerable bearing on the size of initial cohort required. Consequently, the sample size calculation must necessarily estimate not only the numbers for the cmRCT itself, but also the size of required cohort, to be certain that meeting the sample requirement for the cmRCT is feasible. Existing cmRCTs have not considered this and we believe that actual required cohort sizes will in many cases be much larger than what is commonly assumed. This is particularly important because increasing the size of the cohort later is usually not viable. This again emphasizes the importance of pilot work to gauge levels of consent and eligibility before embarking on a full trial. In this article, we have presented a simple method for adjusting a hypothesized effect for anticipated nonconsent, along with guidance for computing sample sizes for both a cmRCT and the host cohort.

A cmRCT faces threats to internal validity that are specific to this design. Because patients not selected for treatment are automatically assigned to the control group, any deviation from a uniform probability of selection can produce a systematic difference between trial arms. It seems easy to go wrong here, such as by failing to include noncontactable patients in top-up samples. Other complexities arise when not all patients can be recruited, or all treatment rolled out, within the same interval between outcome measurements. This causes issues in specifying baselines and in maintaining equivalence between treatment and control arms. Studies using this design need to publish sufficient detail on their processes, along with summary statistics, to reassure users of the research that such validity threats have been appropriately addressed. If this design becomes more widespread, cmRCT-specific Consolidated Standards of Reporting Trials guidance may be indicated.

In summary, the cmRCT research design is an intriguing development that may offer potential advantages over conventional designs. However, a cmRCT is a fundamentally different kind of design to a pRCT and requires tailored power calculation, sampling, and analysis procedures; else, studies may find themselves underpowered or subject to selection and other validity biases. Further research and methodological developments are needed to determine under what conditions and in which contexts a cmRCT represents an optimal design choice.
